# Warwick Mineral Spring

**Published:** 1876-12

**Authors:** 


### *THE WARWICK MINERAL SPRING.*

The Health Food Company have accepted the general agency of the Warwick Mineral Spring, which was discovered some years ago by Mr. James Penrose, of Newark. This fountain has been visited by Prof.

Chandler of this city and Prof. Silliman, of New Haven, both of whom have testified in strong terms to its value as a mineral spring. So long ago as 1870, Prof. Chandler wrote as follows :

School of Mines, Columbia College,<br>New York, Aug. 8, 1870.

Having analyzed the water of the Warwick Spring, and having examined the locality with some care, I am satisfied that the water possesses valuable medicinal properties which will be found to be very important.

It is a genuine mineral spring, of a very peculiar composition, as shown by the analysis. C. F. Chandler, Ph. D.,<br>Prof. of Analytical and Applied Chemistry.

Here is the analysis alluded to :

Columbia College, cor. 49th St. and 4th Ave.,<br>New York, July 7th, 1870.

CERTIFICATE OF ANALYSIS.

Sir: The sample of Mineral Water marked Warwick Spring, submitted to me for examination, contains in one wine gallon of 231 cubic inches :

| | | |
|---|---|---|
| Chloride of Sodium | 13,407 | grains. |
| Chloride of Potassium | 5,400 | " |
| Bromide of Sodium | a trace. | |
| Bicarbonate of Soda | 11,264 | " |
| Bicarbonate of Ammonia | 18,431 | " |
| Bicarbonate of Magnesia | 18,093 | " |
| Bicarbonate of Lime | 41,822 | " |
| Bicarbonate of Iron | 2,722 | " |
| Phosphate of Soda | 0,094 | " |
| Nitrate of Potassa | a trace. | |
| Alumina | 0.700 | " |
| Silica | 0,816 | " |
| Organic Matter | a trace. | |
| Total | 112,749 | grains. |

Carbonic Acid Gas, 101,028 cubic inches.

Respectfully your obedient Servant,<br>C. F. CHANDLER, Ph. D.,<br>Professor of Analytical and Applied Chemistry.

---

Prof. Silliman wrote as follows after visiting the Spring :

The chemical constitution of the Warwick Mineral Spring is clearly established by Prof. Chandler's analysis, to be a chalybeate and saline water, of very remarkable and unusual character. Under proper advice it must prove a valuable agent in a large class of cases.

B. SILLIMAN,<br>Professor of Chemistry, Yale College.

---

Upon the publication of the opinions of these distinguished gentlemen, the water was sought by many sufferers from debility, dyspepsia, diabetes, and headache, and a great array of testimony to its curative value quickly accumulated. In consideration of its now unquestioned excellence, the Health Food Company have taken hold of it with a view to its extensive introduction. It may be found on draught at the office of the Company, where all are invited to test it without expense. It is supplied in cases of 4 dozen pint bottles to such as desire to use it at home.

Circulars, giving the analysis and testimony of medical men and others as to the value of the Warwick Water, are sent free on application.

